# PIAS1 potentiates the anti-EBV activity of SAMHD1 through SUMOylation

**DOI:** 10.1186/s13578-021-00636-y

**Published:** 2021-07-08

**Authors:** Farjana Saiada, Kun Zhang, Renfeng Li

**Affiliations:** 1 School of Dentistry, Philips Institute for Oral Health Research, Virginia Commonwealth University, Richmond, VA 23298 USA; 2 Department of Microbiology and Immunology, School of Medicine, Virginia Commonwealth University, Richmond, VA 23298 USA; 3 Massey Cancer Center, Virginia Commonwealth University, Richmond, VA 23298 USA

**Keywords:** SAMHD1, PIAS1, Restriction factor, Epstein-Barr virus, Cytomegalovirus, SUMOylation, Herpesvirus, Deoxynucleotide triphosphohydrolase, Phosphorylation

## Abstract

**Background:**

Sterile alpha motif and HD domain 1 (SAMHD1) is a deoxynucleotide triphosphohydrolase (dNTPase) that restricts the infection of a variety of RNA and DNA viruses, including herpesviruses. The anti-viral function of SAMHD1 is associated with its dNTPase activity, which is regulated by several post-translational modifications, including phosphorylation, acetylation and ubiquitination. Our recent studies also demonstrated that the E3 SUMO ligase PIAS1 functions as an Epstein-Barr virus (EBV) restriction factor. However, whether SAMHD1 is regulated by PIAS1 to restrict EBV replication remains unknown.

**Results:**

In this study, we showed that PIAS1 interacts with SAMHD1 and promotes its SUMOylation. We identified three lysine residues (K469, K595 and K622) located on the surface of SAMHD1 as the major SUMOylation sites. We demonstrated that phosphorylated SAMHD1 can be SUMOylated by PIAS1 and SUMOylated SAMHD1 can also be phosphorylated by viral protein kinases. We showed that SUMOylation-deficient SAMHD1 loses its anti-EBV activity. Furthermore, we demonstrated that SAMHD1 is associated with EBV genome in a PIAS1-dependent manner.

**Conclusion:**

Our study reveals that PIAS1 synergizes with SAMHD1 to inhibit EBV lytic replication through protein–protein interaction and SUMOylation.

## Background

Host restriction factors serve as the first line of defense against viral infection through blocking virus entry, replication or release. One recently discovered restriction factor is the Sterile alpha motif and HD domain 1 (SAMHD1) protein, which hydrolyzes deoxyribonucleoside triphosphates (dNTPs) to reduce the cellular dNTP pool required for viral infection and propagation. In addition to limiting human immunodeficiency virus-1 (HIV-1) infection [1], SAMHD1 has also been shown to restrict the infection of herpesviruses [Epstein-Barr virus (EBV), human/mouse cytomegalovirus (HCMV/MCMV) and human simplex virus 1 (HSV-1) [2–7], vaccinia virus [2], human T cell leukemia virus type 1 [8], hepatitis B virus [9] and human papillomavirus 16 [10].

The anti-viral activity of SAMHD1 can be counterbalanced by different viral proteins. HIV-2/SIV virion-associated Vpx accessory proteins bind to SAMHD1 and E3 ubiquitin ligase complex to promote SAMHD1 ubiquitination and proteasome-dependent degradation [11–13]. Phosphorylation of SAMHD1 by the conserved herpesvirus protein kinases [4–7] and cellular kinases CDK1 and CDK2 diminishes its anti-viral activity [3, 14–16]. Although earlier studies suggested that SAMHD1’s deoxynucleotide triphosphohydrolase (dNTPase) activity is not regulated by phosphorylation, recent studies revealed that phosphorylation selectively suppress its dNTPase activity to increase the dNTP level for optimal viral DNA synthesis [6, 17–22]. Phosphorylation of SAMHD1 has also been implicated in its cytoplasmic re-localization during HCMV infection [16]. In addition, SAMHD1 is also acetylated on K405 by ARD1 to enhance its dNTPase activity [23].

Protein SUMOylation is mediated by a series of enzymes that catalyze the transfer of small ubiquitin-related modifier (SUMO) to a protein substrate. These enzymes are consist of an E1 activating enzyme complex SAE1/UBA2, an E2 conjugating enzyme UBC9 and an E3 protein ligase. E3 SUMO ligases bind to specific target proteins to determine its substrate specificity [24]. Protein SUMOylation plays important roles in diverse biological processes, including gene transcription, DNA replication, and viral infection [24, 25]. With the advances in mass spectrometry, recent studies identified thousands of SUMOylation sites within the human proteome [26–28].

In contrast to ubiquitination, there are only a few E3 ligases for SUMOylation. The protein inhibitor of STAT (PIAS) family proteins are a group of RING domain-containing E3 SUMO ligases responsible for the transfer of SUMO proteins from UBC9 to the protein substrates. The PIAS family proteins consist of PIAS1, PIASx/PIAS2, PIAS3 and PIASy/PIAS4 with their unique substates [29]. PIAS1 restricts HSV-1 infection when ICP0 is knocked out [30]. Our group recently also identified PIAS1 as an EBV restriction factor, which is cleaved by caspases upon lytic induction [31]. PIAS1 can trigger the SUMOylation of a group of cellular and viral proteins, including p53 [32–35] and EBV immediate-early protein RTA [36].

In this study, we demonstrated that PIAS family members PIAS1, PIAS2, PIAS3 and PIAS4 interact with SAMHD1 and PIAS1 possesses the capability to promote SAMHD1 SUMOylation. We identified K469, K595 and K622 as the major SUMOylation sites on SAMHD1. We showed that PIAS1 promotes the anti-EBV activity of SAMHD1 in a SUMOylation dependent manner, which expands our understanding of SAMHD1 regulation by post-translational modifications.

## Results

### PIAS family proteins interact with SAMHD1

**Fig. 1:**
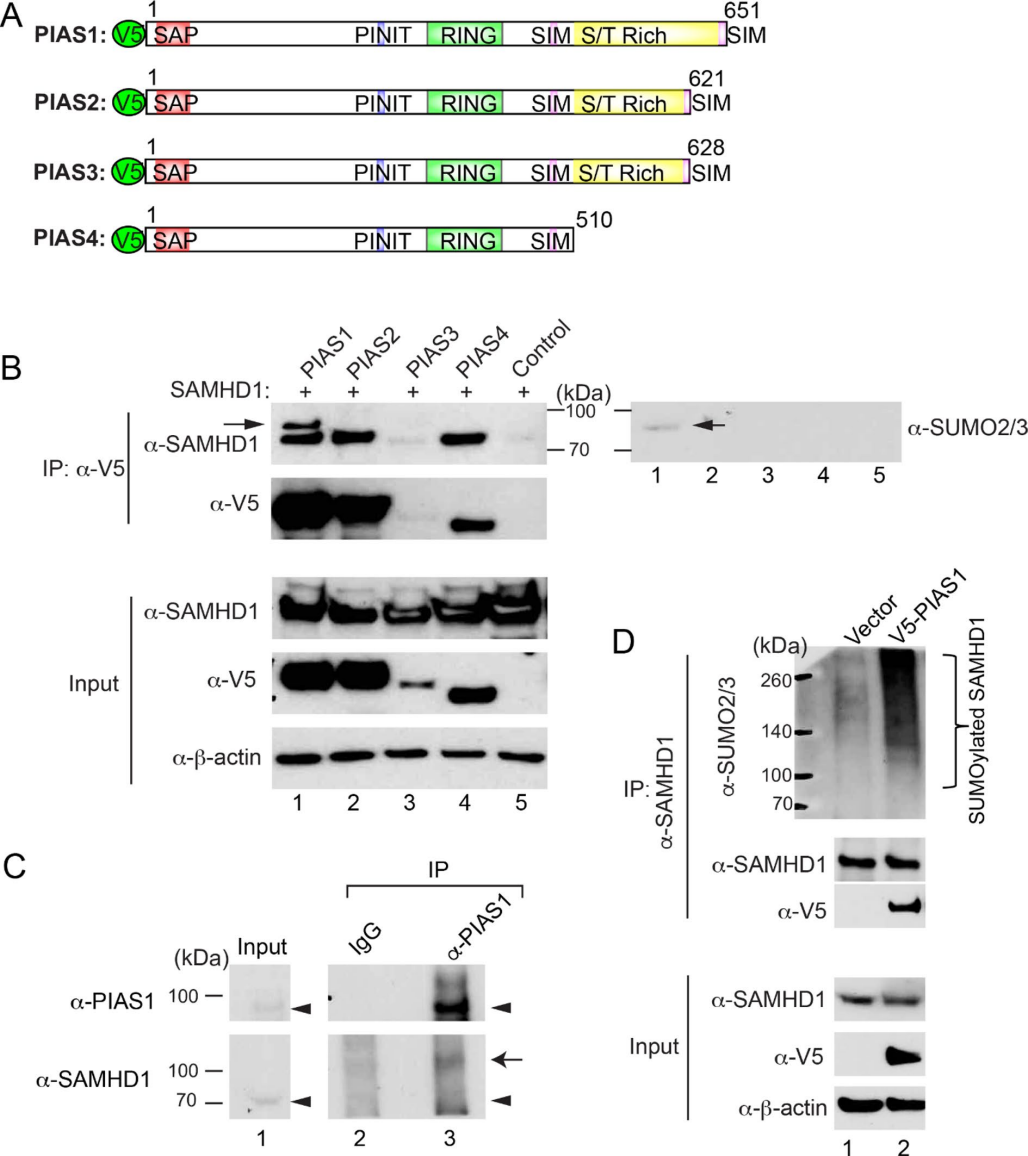
PIAS family proteins interact with SAMHD1. **A** Schematic representation of V5-Tagged PIAS family proteins. SAP (SAF-A/B, Acinus, and PIAS): DNA and protein binding domain; PINIT: nuclear localization motif; RING finger: E3 ligase domain for protein SUMOylation; SIM: SUMO interacting motif; S/T Rich: variable Ser/Thr rich region. **B** PIAS1, PIAS2, and PIAS4 interact with SAMHD1. WB analysis showing Co-IP of SAMHD1 with PIAS proteins using 293 T cells transfected with SAMHD1 and individual PIAS proteins as indicated. Input, 2% whole-cell lysate used for IP. Left arrow denotes modified SAMHD1 in the PIAS1 and SAMHD1 co-transfection group. Anti-SUMO2/3 antibody was used to show a SUMOylated band as indicated (right arrow). **C** PIAS1 interacts with SAMHD1 in vivo. Endogenous PIAS1 was immunoprecipitated by anti-PIAS1 antibody or rabbit IgG control using cell lysate from 293 T cells. The Co-IP of SAMHD1 was examined by WB analysis. The positions of PIAS1 and SAMHD1 were labeled by arrowheads. Arrow denotes modified SAMHD1. **D** PIAS1 promotes SAMHD1 SUMOylation. Endogenous SAMHD1 was immunoprecipitated from cells transfected with vector control or PIAS1. WB was performed using anti-SUMO2/3 or other antibodies as indicated. Bracket denotes SUMOylated SAMHD1

Our previous studies have demonstrated that both PIAS1 and SAMHD1 restrict EBV lytic replication [6, 31]. The nuclear localization of PIAS1 and SAMHD1 suggest that these two proteins may interact with each other to block viral replication. To determine whether PIAS1 and/or other members of the PIAS family interact with SAMHD1 (Fig. 1A), we co-transfected SAMHD1 with individual PIAS construct into 293 T cells and performed co-immunoprecipitation (Co-IP) experiments (Fig. 1B). We found that SAMHD1 is strongly Co-IPed by PIAS1, PIAS2, PIAS4 (Fig. 1B**, **lanes 1, 2 and 4). The weaker Co-IP signal of SAMHD1 by PIAS3 is possibly due to PIAS3 protein expression level (Fig. 1B, lanes 3). Interestingly, we also observed an extra band when SAMHD1 is Co-IPed by PIAS1 (Fig. 1B, lane 1), suggesting that PIAS1 may strongly interact with a modified SAMHD1. Because PIAS1 contain two SUMO interacting motifs (SIM) that can bind to SUMOylated proteins [37], the modified SAMHD1 may represent a SUMOylated form with a molecular weight of approximately 10 kDa higher than SAMHD1. Indeed, we found that there is a band in the same position as modified SAMHD1 using anti-SUMO2/3 antibody, suggesting this Co-IPed SAMHD1 is a SUMOylated form (Fig. 1B, SUMO2/3 blot). To further determine whether PIAS1 interacts with SAMHD1 in vivo, we immunoprecipitated endogenous PIAS1 by anti-PIAS1 antibody using cell lysate from 293 T cells. We found that a higher molecular weight of SAMHD1 is being pulled down, suggesting that PIAS1 interacts with modified SAMHD1 in vivo (Fig. 1C).

### PIAS1 promotes the SUMOylation of SAMHD1

Because PIAS1 serves as an E3 SUMO ligase for both viral and cellular proteins, we reasoned that PIAS1 could trigger the SUMOylation of SAMHD1. To test this possibility, we immunoprecipitated endogenous SAMHD1 from cells transfected with vector control or PIAS1 and performed Western Blot (WB) using anti-SUMO2/3 antibody. We found that SAMHD1 poly-SUMOylation level is significantly higher in PIAS1-expressing cells than that in the vector control cells (Fig. 1D).

**Fig. 2:**
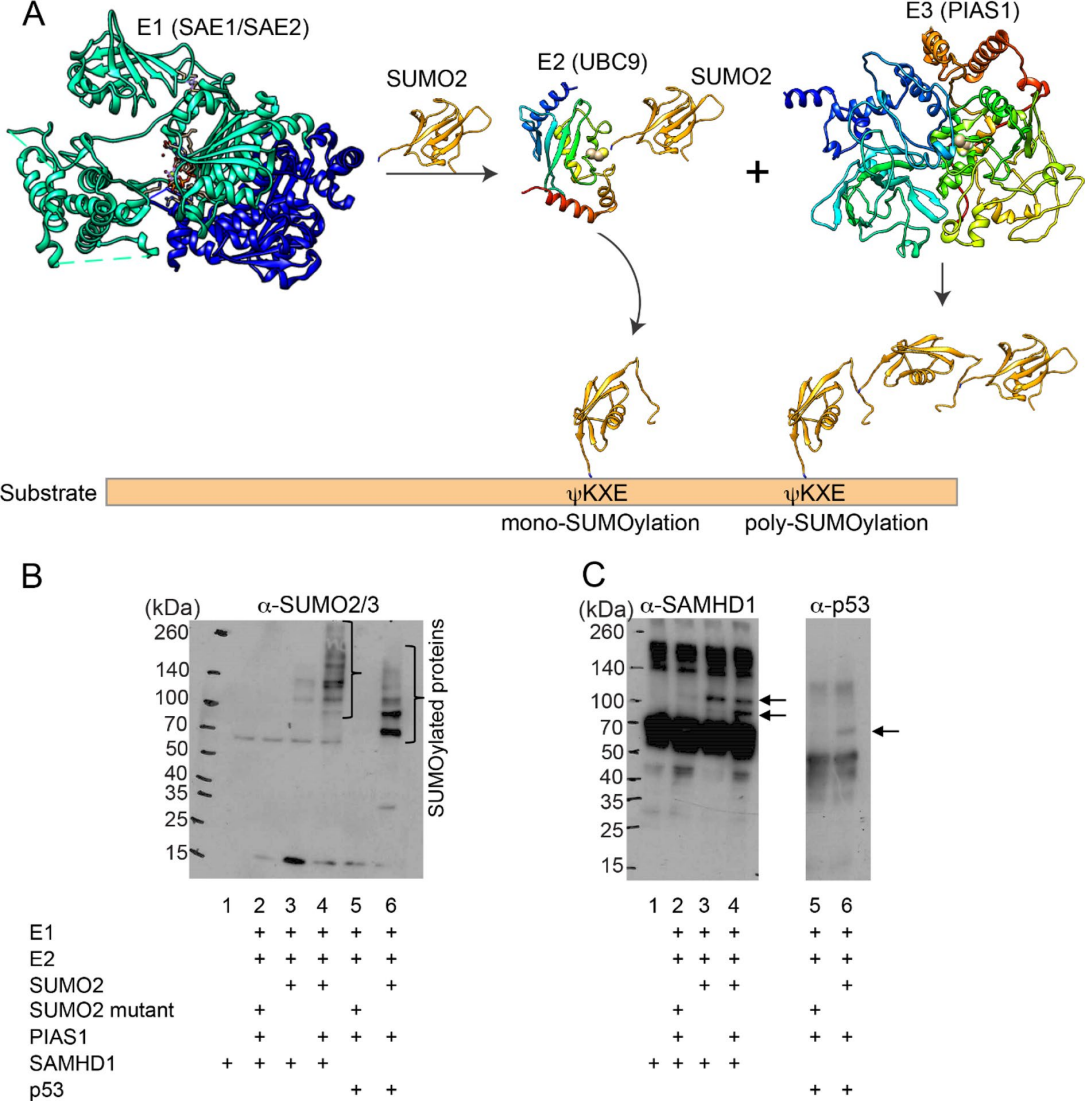
PIAS1 promotes the SUMOylation of SAMHD1. **A** Schematic representation of protein SUMOylation process. The process involves E1 (PDB# 1Y8Q), E2 (PDB# 1A3S), SUMO2 (PDB# 2N1W), PIAS1 [modelled structure from I-TASSER, see reference [31] and protein substrate. Mono- and poly-SUMOylation were labeled as indicated. (**B**, **C**) PIAS1 promotes SAMHD1 SUMOylation. In vitro SUMOylation assay was performed with the combination of E1, E2, SUMO2, SUMO2 mutant, PIAS1, and substrates (SAMHD1 and p53) as indicated. The reaction was terminated with SDS sample loading buffer and WB was performed using anti-SUMO2/3 (**B**) or anti-SAMHD1 and anti-P53 (**C**) antibodies. Brackets denote SUMOylated SAMHD1 and p53. Arrows denote mono- or di-SUMOylated SAMHD1 and p53

To further confirm these results, we performed in vitro SUMOylation assay with recombinant proteins (Fig. 2A). We first performed Western Blot (WB) using anti-SUMO2/3 antibody (Fig. 2B). We found that SAMHD1 is SUMOylated when incubated with E1, E2 and wild-type (WT) SUMO2 (Fig. 2B, lane 3) but not with SUMO2 mutant (Fig. 2B, lane 2). We observed that PIAS1 further promotes SAMHD1 poly-SUMOylation with WT SUMO2 but not SUMO2 mutant (Fig. 2B, lane 4 vs 2). As a positive control, we also found that PIAS1 triggers the SUMOylation of p53 with WT SUMO2 but not SUMO2 mutant (Fig. 2B, lane 6 vs 5). To further confirm these results, we used anti-SAMHD1 antibody for WB and found that PIAS1 indeed promotes the SUMOylation of both SAMHD1 and p53 (Fig. 2C, lanes 4 and 6).

### PIAS1 synergizes with SAMHD1 to restrict EBV replication

**Fig. 3:**
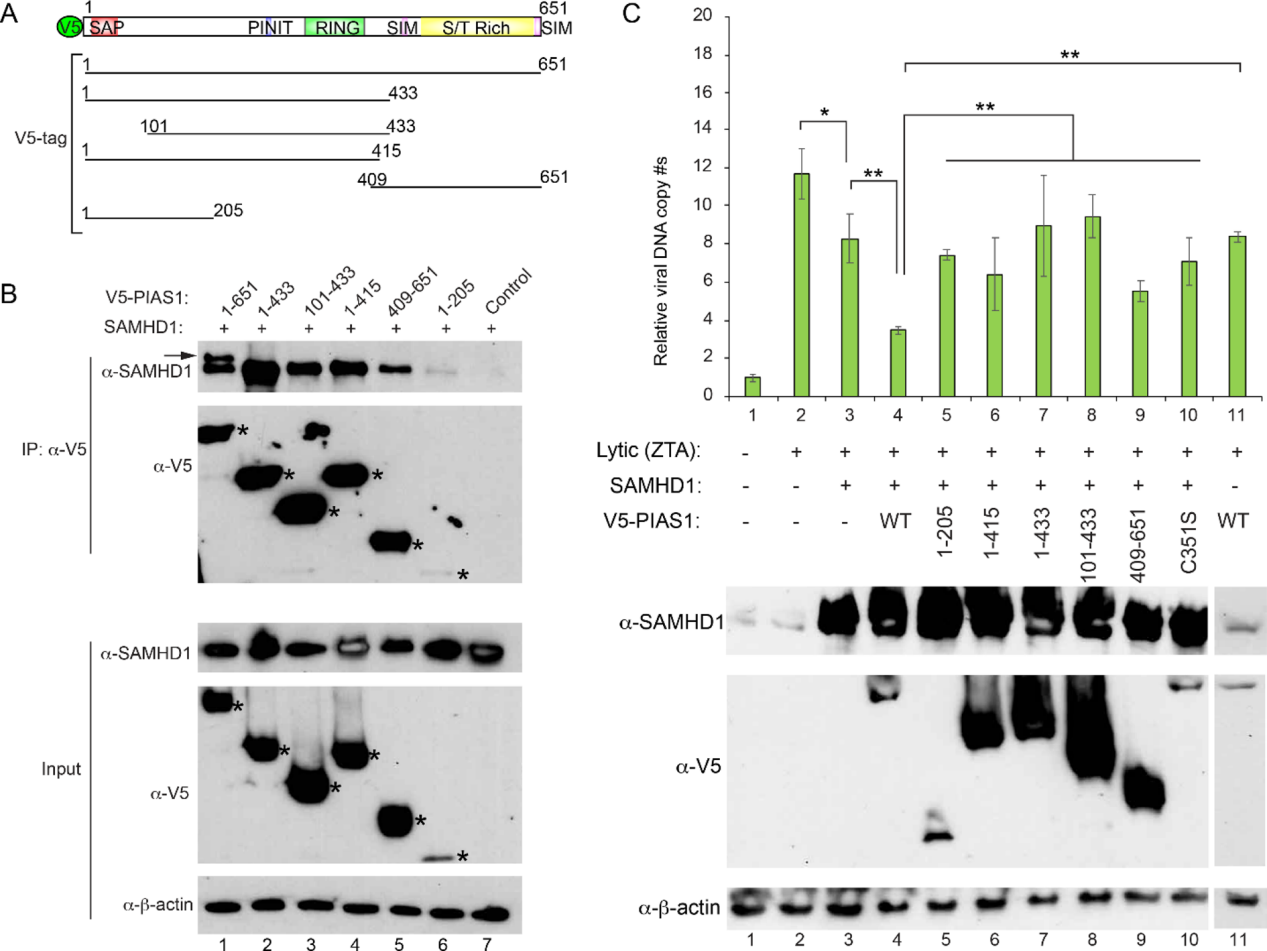
PIAS1 synergizes with SAMHD1 to restrict EBV replication. **A** The schematic representation of full length PIAS1 (1–651) or PIAS1 truncation mutants. **B** The central and C-terminal part of PIAS1 interacts with SAMHD1. 293 T cells were co-transfected with SAMHD1 and V5-tagged full length PIAS1 (aa 1–651) or PIAS1 truncation mutants as indicated. WB analyses showing that SAMHD1 is Co-IPed with the central and C-terminal part of PIAS1. Co-IP, co-immunoprecipitation; β-actin blot was included as loading controls. Arrow denotes modified SAMHD1 in the PIAS1 and SAMHD1 co-transfection group. **C** Full-length PIAS1 synergizes with SAMHD1 in restricting EBV replication. HEK293 (EBV +) cells were co-transfected with plasmid DNA encoding ZTA (lytic trigger), SAMHD1, and full-length (WT), truncated PIAS1 (1–205, 1–415, 1–433 and 409–651) or E3 ligase-deficient (C351S) mutant as indicated. The relative EBV copy numbers were measured using the qPCR as described in the method. The expression levels of SAMHD1 and PIAS1 were monitored by WB. β-actin blot was included as loading controls. Results from three biological replicates are presented. Error bars indicate the standard deviation. * p < 0.05, ** p < 0.01

To determine the regions of PIAS1 responsible for binding to SAMHD1, we co-transfected SAMHD1 with our previously created PIAS1 truncation mutants into 293 T cells (Fig. 3A, B). We found that all the PIAS1 truncation mutants (aa 1–433, 101–433, 1–415 and 409–651) all bind to SAMHD1 but only full-length PIAS1 can pull down the modified SAMHD1 (Fig. 3B, lanes 1–5). PIAS1 (aa 1–205) showed less binding to SAMHD1 possibly due to the reduced expression level (Fig. 3B, lane 6).

To demonstrate whether PIAS1 and SAMHD1 association plays a role in EBV lytic replication, we transfected 293 (EBV +) cells with vectors expressing ZTA (a trigger for EBV lytic reactivation), SAMHD1 and different PIAS1 truncation mutants. As expected, transfection of SAMHD1 or PIAS1 only partially reduced EBV lytic replication (Fig. 3C, lane 2 vs 3 and 11). Interestingly, we found that co-transfection of WT PIAS1 and SAMHD1 greatly suppresses EBV DNA replication (Fig. 3C, lane 4). However, PIAS1 truncation mutants or E3 ligase deficient mutant (C351S) failed to synergize with SAMHD1 in blocking viral replication (Fig. 3C, lanes 4 vs 5–10). Together, these results suggested that PIAS1-mediated SAMHD1 SUMOylation plays a role in limiting EBV replication.

### PIAS1 promotes SAMHD1 SUMOylation

**Fig. 4:**
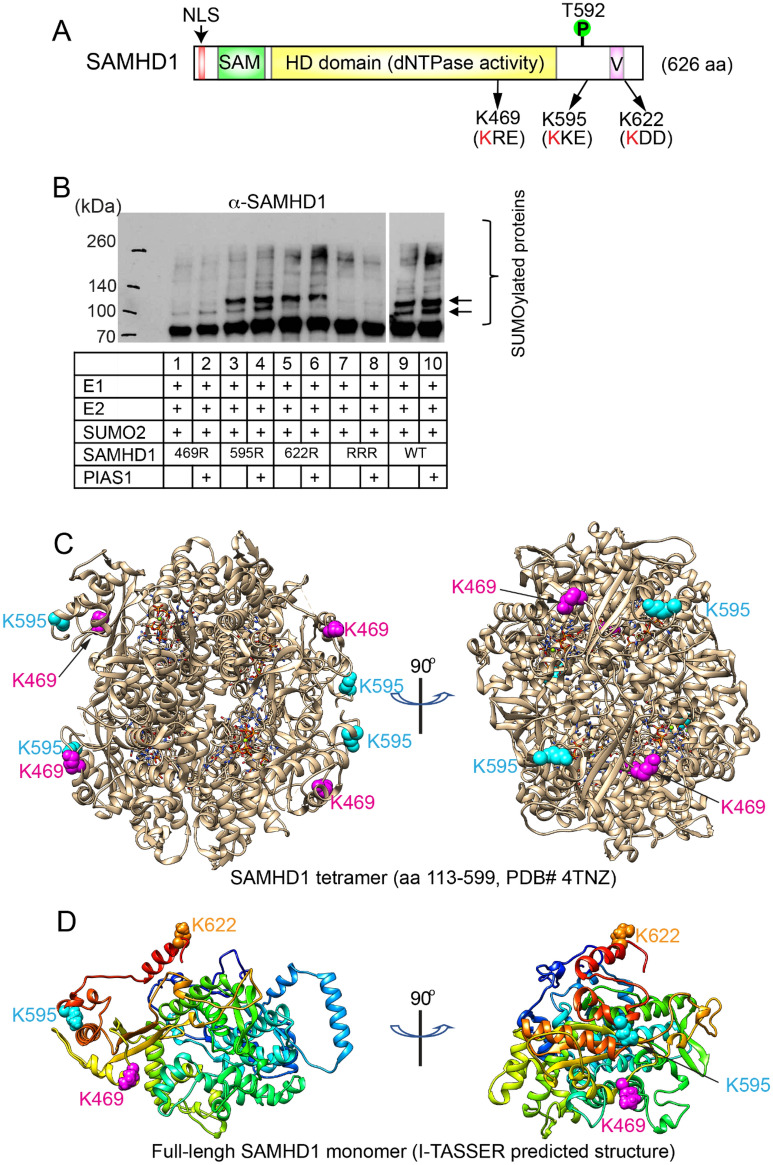
PIAS1 SUMOylates SAMHD1 at K469, K595, and K622. **A** The schematic representation of SAMHD1 protein (1–626). The three SUMOylation consensus motifs were labeled as indicated. *NLS* nuclear localization signal, *SAM* sterile alpha motif, *HD domain* histidine and aspartic acid domain, *V* vpx interacting motif; T592: phosphorylation site mediated by cellular CDK1/2 and the conserved herpesvirus protein kinases. **B** In vitro SUMOylation assay was performed with the combination of E1, E2, SUMO2, PIAS1, and WT or individual SAMHD1 mutant [K469R, K595R, K622R, or RRR(K469R/K595R/K622R)] as indicated. The reaction was terminated with SDS sample loading buffer and WB was performed using anti-SAMHD1. Arrows denote mono- or di-SUMOylated SAMHD1. **C** The localization of K469 and K595 in SAMHD1 tetramer. The SAMHD1 tetramer structure (PDB# 4TNZ) was used to locate K469 and K595 using Chimera software. **D** The localization of K469, K595 and K622 in full-length SAMHD1 monomer. The SAMHD1 structure was predicted using I-TASSER algorithm and localization of K469, K595 and K622 was labeled using Chimera software

To identify the SUMOylation sites on SAMHD1, we searched the mass spectrometry database on protein SUMOylation and found that three sites (K469, K595, K622) within SAMHD1 protein are SUMOylated in Hela cells [26–28]. These sites are located within the typical KxE/D motif that is required for SUMOylation (Fig. 4A).

To determine whether these are the SAMHD1 SUMOylation sites, we mutated these sites individually and in combination to generate four SAMHD1 mutants, namely K469R, K595R, K622R and RRR (K469R/K595R/K622R). We then purified these proteins and performed in vitro SUMOylation experiments. We found that when K469 is mutated, the upper band (~ 100 kDa) is disappeared, suggesting that the upper band mainly derives from SUMOylation at K469 (Fig. 4B, lanes 1–2 vs 9–10). For K469R, K595R, and K622R mutants, the lower band intensity was reduced in K622R, and to a lesser extent K469R and K595R mutants, compared with WT SAMHD1 (Fig. 4B, lanes 3–8 vs 9–10), suggesting that the lower band derives from SUMOylation at multiple sites, especially K622. The RRR mutant lost all the major SUMOylation bands detected by anti-SAMHD1 antibody (Fig. 4B, lanes 7–8 vs 9–10), suggesting these three sites are the major SUMOylation site on SAMHD1 mediated by PIAS1.

Based on the tetrameric structure of SAMHD1 obtained by X-ray crystallography [38], we found that both K469 and K595 are located on the surface the protein (Fig. 4C). Because K622 is located at the very C-terminal of SAMHD1 and is not part of the solved structure, we used I-TASSER algorithm to predict the full-length SAMHD1 structure and found that K622 is also located on the surface of the protein (Fig. 4D). We reasoned that the surface localization of these lysine residues is favorable for SUMOylation.

### Phosphorylation does not affect SAMHD1 SUMOylation and SUMOylated SAMHD1 can be phosphorylated by viral protein kinases

**Fig. 5:**
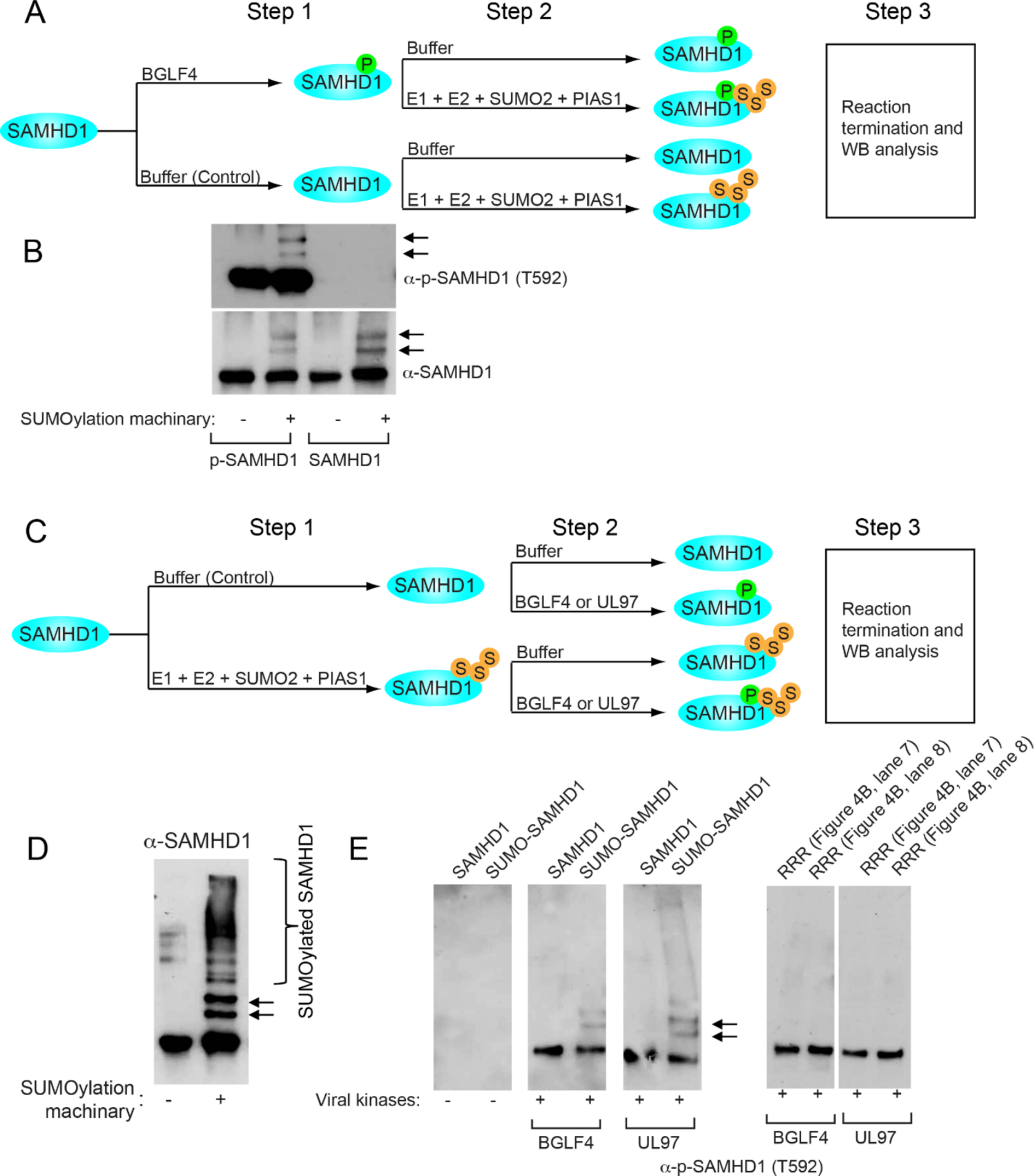
Phosphorylation enhances SAMHD1 SUMOylation and SUMOylated SAMHD1 can be phosphorylated by viral protein kinases. **A** Schematic representation of SAMHD1 phosphorylation and subsequent SUMOylation analysis. Step 1: Recombinant SAMHD1 was phosphorylated by EBV protein kinase BGLF4 in vitro. Non-phosphorylated SAMHD1 was similarly processed by incubating with Buffer control. Step 2: The phosphorylated and non-phosphorylated SAMHD1 were used to perform in vitro SUMOylation reaction using E1, E2, SUMO2 and PIAS1 or buffer control. Step 3: The reaction was terminated by adding 2 × SDS sample loading buffer and the samples were analyzed by WB. **B** Phosphorylated and non-phosphorylated SAMHD1 generated in (**A**) Step 1 were incubated with buffer control or with SUMOylation machinery. The SUMOylation and phosphorylation of SAMHD1 were analyzed by WB using anti-SAMHD1 and anti-p-SAMHD1 (T592) antibodies as indicated. Arrows denote mono-or di-SUMOylated SAMHD1. **C** Schematic representation of SAMHD1 SUMOylation and subsequent phosphorylation analysis. Step 1: Recombinant SAMHD1 was SUMOylated by E1, E2, SUMO2 and PIAS1 in vitro. Non-SUMOylated SAMHD1 was similarly processed by incubating with buffer control. Step 2: The SUMOylated and non-SUMOylated SAMHD1 were subjected to in vitro kinase reaction using viral protein kinases (EBV BGLF4 or HCMV UL97). **D** WB analysis showing that SUMOylated SAMHD1 was generated from (**C**) Step 1. Arrows and bracket denote mono- and poly-SUMOylated SAMHD1, respectively. **E** SUMOylated and non-SUMOylated SAMHD1 generated in (**C**) Step 1 were incubated with buffer control or with EBV BGLF4 and HCMV UL97 in step 2. As controls, SAMHD1-RRR mutant samples from Fig. 4B (lanes 7 and 8) were also incubated with EBV BGLF4 and HCMV UL97. The phosphorylation status was analyzed by WB using anti-p-SAMHD1 (T592) antibodies. Arrows denote mono- or di-SUMOylated SAMHD1

SAMHD1 can be phosphorylated by the conserved herpesvirus protein kinases and cellular kinases CDK1 and CDK2 on T592 [6]. To determine whether SAMHD1 phosphorylation affects its SUMOylation, we generated phosphorylated SAMHD1 using EBV protein kinase BGLF4 (Fig. 5A). The phosphorylated and non-phosphorylated SAMHD1 were then subjected to in vitro SUMOylation reactions. We found that phospho-SAMHD1 is still SUMOylated by PIAS1 and its SUMOylation level is similar to that of non-phospho-SAMHD1 (Fig. 5B).

To determine whether SAMHD1 SUMOylation affects its phosphorylation, we generated SUMOylated SAMHD1 using E1, E2, SUMO2 and PIAS1 (Fig. 5C). The SUMOylated and non-SUMOylated SAMHD1 were then subjected to in vitro phosphorylation reactions. We found that SUMOylated SAMHD1 is still phosphorylated by the conserved herpesvirus protein kinases, including EBV BGLF4 and HCMV UL97 (Fig. 5D–E). In addition, we also observed that SAMHD1-RRR mutant can also be phosphorylated by viral protein kinases (Fig. 5E).

These results together suggested that phosphorylation does not affect SAMHD1 SUMOylation and SUMOylation does not block SAMHD1 phosphorylation by viral protein kinases.

### SUMOylation promotes the anti-viral activity of SAMHD1

**Fig. 6:**
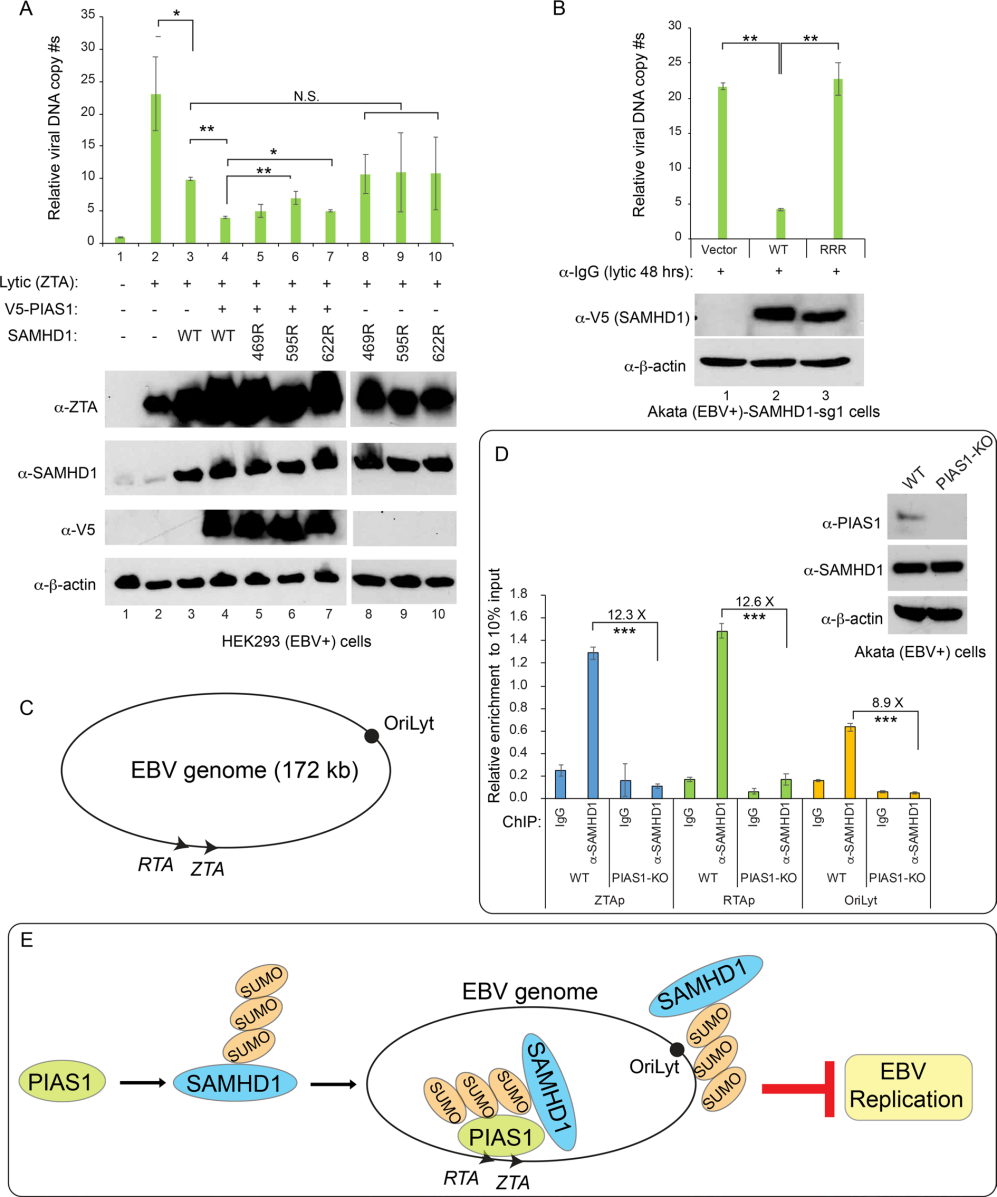
SUMOylation-deficient SAMHD1 impairs its anti-viral activity. **A** SUMOylation-deficient SAMHD1 impairs its anti-EBV activity. HEK293 (EBV +) cells were co-transfected with plasmid DNA encoding ZTA (lytic trigger), WT PIAS1, and WT or mutant SAMHD1 as indicated. The relative EBV copy numbers were measured using the qPCR as described in the method. The expression levels of ZTA, SAMHD1 and PIAS1 were monitored by WB. β-actin blot was included as loading controls. Results from three biological replicates are presented. Error bars indicate the standard deviation. *p < 0.05, **p < 0.01. **B** Akata (EBV +)-SAMHD1-sg1 cells (endogenous SAMHD1 is depleted by CRISPR/Cas9) were used to create cell lines using vector control (pLX304), WT SAMHD1 (pLX-304-V5-SAMHD1), and SUMOylation-deficient SAMHD1 [pLX-304-V5-SAMHD1-(RRR)]. EBV lytic cycle was induced by anti-IgG-mediated B cell receptor cross-linking. The relative EBV copy numbers were measured using the qPCR as described in the method. The expression levels of SAMHD1 were monitored by WB using anti-V5 antibody. β-actin blot was included as loading controls. Results from three biological replicates are presented. Error bars indicate the standard deviation. *p < 0.05, ** p < 0.01. **C** The relative positions of EBV ZTA, RTA and OriLyt are labeled as indicated. **D** PIAS1 regulates SAMHD1 association with EBV genome. ChIP-PCR analysis performed on Akata (EBV +) cells showing SAMHD1 binding to ZTA (ZTAp) and RTA (RTAp) promoters and OriLyt region in WT control cells but not PIAS1-knockout (PIAS1-KO) cells. ChIP by a nonspecific IgG was included as a negative control. The expression levels of PIAS1, SAMHD1 and β -actin were monitored by WB. Results from three biological replicates are presented. Error bars indicate the standard deviation. ***p < 0.001. **E** Hypothesized model. PIAS1 regulates the SUMOylation and viral genome recruitment of SAMHD1 to restrict EBV lytic replication

To determine the function of SAMHD1 SUMOylation in EBV replication, we transfected 293 (EBV +) cells with vectors expressing PIAS1, WT and SUMOylation-deficient SAMHD1 mutants. Without PIAS1 co-transfection, WT SAMHD1 and K469R, K595R and K622R mutants displayed similar anti-EBV activity (Fig. 6A, lanes 4 and 8–10). With PIAS1 co-transfection, SUMOylation-deficient mutants, especially K595R, displayed higher viral replication compared to those with WT SAMHD1 (Fig. 6A, lanes 5, 6, 7 vs 4), suggesting SUMOylation promotes the anti-EBV activity of SAMHD1.

To further confirm these results in EBV-naturally infected cells, we used Akata EBV ( +) cells with SAMHD1 depleted by CRISPR/Cas9 genomic editing [6]. We reconstituted the cells with WT and SUMOylation-deficient SAMHD1 (RRR, K469R/K595R/K622R). We then induced EBV replication with anti-IgG mediated cross-linking of B cell receptor. We found that EBV replication is higher in cells expressing SUMOylation-deficient SAMHD1 compared with cells expressing WT SAMHD1 (Fig. 6B).

PIAS1 binds to EBV genome and inhibits EBV lytic gene expression [31, 39]. We hypothesized that PIAS1 may recruit SAMHD1 to EBV genome. To test this hypothesis, we compared SAMHD1 binding to EBV genome [ZTA and RTA promoter regions and EBV lytic replication origin (OriLyt)] in wild-type and PIAS1-knockout Akata (EBV +) cells that we established previously [31]. We found that SAMHD1 is enriched in these regions within the EBV genome in wild-type Akata (EBV +) cells, but this enrichment is abolished when PIAS1 is knockout (Fig. 6C, D).

Together, our results suggested that PIAS1 promotes the anti-EBV activity of SAMHD1 through SUMOylation and SAMHD1 enrichment in EBV genome (Fig. 6E).

## Discussion

Post-translational modifications have been implicated in regulating the diverse aspects of protein function. The advancement in mass spectrometry field has greatly contributed to the discovery of many post-translational modifications, including phosphorylation, ubiquitination, acetylation and SUMOylation [26–28, 40–42]. Phosphorylation, acetylation, and ubiquitination all have been reported in the regulation of SAMHD1’s stability and/or anti-viral activity [6, 15, 17, 19, 21, 23, 43–45]. In this study, we demonstrated that SAMHD1 is SUMOylated and its anti-viral activity is enhanced by PIAS1-mediated SUMOylation.

As a cellular dNTPase, SAMHD1 restricts a wide range of RNA and DNA viruses, including HSV and EBV [1–3, 9]. Depletion of the dNTPs pool is considered as the primary anti-viral mechanism for SAMHD1. However, studies in HIV-1 infection suggested an anti-viral role beyond its dNTPase activity [15], indicating that SAMHD1 might be regulated by other cofactors or post-translational modifications.

Recently, we and others demonstrated that the E3 SUMO ligase PIAS1 restricts the replication of EBV and ICP0-null HSV-1 [30, 31, 46]. In this study, we showed that PIAS1 and other PIAS family proteins interact with SAMHD1 and PIAS1 can promote SAMHD1 SUMOylation. We mapped the binding regions between SAMHD1 and PIAS1 and discovered that only full-length PIAS1 has the capability to pull down SUMOylated SAMHD1. Although C-terminal part of PIAS1 contain two SIMs [37], it did not pull down SUMOylated SAMHD1, suggesting that the local structure or orientation of two SIMs might be affected by truncations. Alternatively, SAMHD1 might be SUMOylated during the Co-IP process, which requires a full-length functional PIAS1.

We demonstrated that PIAS1 synergizes with SAMHD1 to restrict EBV DNA replication while PIAS1 truncation or SUMO ligase-deficient mutants failed to do so, indicating that PIAS1-mediated SUMOylation enhances the anti-viral activity of SAMHD1. In addition to PIAS1, other PIAS family members also interact with SAMHD1 and, therefore, may contribute to the anti-viral activity of SAMHD1 via SUMOylation.

Using in vitro SUMOylation assays, we discovered three major SUMOylation sites located on the surface of SAMHD1. The mutation of these three sites did not completely block SAMHD1 SUMOylation, implying the existence of other minor SUMOylation sites. The in vitro SUMOylation assay kit we used in this study contains SUMO2 but not SUMO1. It is highly possible that SAMHD1 can be modified by SUMO1 on the same sites. We also did not take the oligomerization status of SAMHD1 into consideration for in vitro SUMOylation assay. Therefore, it is possible that monomeric, dimeric, and tetrameric SAMHD1 may display different SUMOylation profile.

Because phosphorylation on T592 was reported to antagonize SAMHD1’s anti-viral activity [6, 15, 17, 19, 21, 43], we also tested whether SUMOylation and phosphorylation could affect each other. We found that phosphorylated SAMHD1 can still be SUMOylated by PIAS1, suggesting that SUMOylation could have an additional layer of regulation. Furthermore, when SAMHD1 is pre-SUMOylated, we found that it can also be phosphorylated by EBV BGLF4 and HCMV UL97. We also observed that in reaction mixture containing non-SUMOylated SAMHD1 and SUMOylated SAMHD1, the phosphorylation of non-SUMOylated SAMHD1 is reduced (Fig. 5). Because BGLF4 is a SUMO binding protein [25], it may prefer to bind to SUMOylated SAMHD1 in the presence of both SUMOylated and non-SUMOylated proteins.

To determine whether SAMHD1 SUMOylation plays any role in EBV replication, we created a series of SUMO-deficient SAMHD1 mutants. We demonstrated that while WT SAMHD1 processes the strong anti-viral activity, SUMO-deficient SAMHD1 mutants display a reduced anti-viral activity. The reconstitution of SAMHD1-knockout cells with WT and K469R/K595R/K622R mutant SAMHD1 further demonstrated that SUMOylated SAMHD1 has higher anti-EBV activity. It was shown that SAMHD1 is ubiquitinated on K622 by TRIM21 [45]. Therefore, SUMOylation on the same sites could theoretically block the ubiquitination and subsequent degradation of SAMHD1.

According to a recent preprint, SAMHD1 was also shown to be SUMOylated on these three sites and SUMOylation also promotes SAMHD1’s anti-HIV activity [47]. Because the SUMOylation-deficient mutations do not affect the dNTPase activity of SAMHD1, the exact role of SUMOylation in SAMHD1’s anti-HIV activity is not clear [47]. We demonstrated that SAMHD1 is enriched in EBV chromatin/genome and this enrichment is dependent on PIAS1. Because PIAS1 contains two SIMs [37], it could recruit SUMOylated SAMHD1 to viral genome. On the other hand, SAMHD1 has also been shown to bind to SUMO through a SIM motif [47]. Therefore. SUMOylated PIAS1 and other chromatin-associated proteins could also serve as a bridge to recruit SAMHD1 to the viral genome. The PIAS1-mediated SAMHD1 enrichment could reduce the local dNTP pool to limit viral DNA replication. In addition, SUMOylated SAMHD1 may have additional function in DNA damage response and immune response critical for blocking viral replication [48, 49]. SUMOylation could also block the acetylation of SAMHD1 to alter its anti-viral activity. Future studies are warranted to explore these possibilities.

## Conclusion

In summary, our data suggest that SAMHD1 is SUMOylated on three major sites and PIAS1 promotes SAMHD1’s anti-EBV activity through enhancing the SUMOylation and viral genome association of SAMHD1. These studies lay a molecular foundation to further explore the regulation of SAMHD1 by PIAS1 and protein SUMOylation.

## Material and methods

### Cell lines and cultures

All Akata (EBV +)-derived cells were grown in RPMI 1640 media supplemented with 10% FBS (Cat# 26,140,079, Thermo Fisher Scientific) in 5% CO_2_ at 37 °C [25, 31, 39, 40, 51]. HEK293 (EBV +) cells carrying B95.8 EBV genome [50, 52] and 293 T cells were grown in DMEM media supplemented with 10% FBS in 5% CO_2_ at 37 °C. (Table 1)

**Table 1:** Key reagents and resources

Reagent or resource	Source	Identifier
Antibodies and reagents		
Anti-phospho-SAMHD1-T592	Cell Signaling Tech	Cat# 15,038
Anti-SAMHD1	Bethyl	Cat# A311-354
Anti-HA	Roche	Cat# 11–867-431–001
Anti-HA-HRP	Cell Signaling Tech	Cat# 14,031
SUMO2 Conjugation Kit	UBPBio	Cat# J3120
SUMOlink SUMO2/3 Kit	Active motif	Cat# 40,220 (discontinued)
Anti-V5 magnetic beads	MBL	Cat# M167-11
Anti-SUMO2/3	MBL	Cat# M114-3
Anti-SUMO2/3	Active motif	Cat# 101,898
Anti-V5-HRP	Thermo Fisher	Cat# R961-25
Anit-V5	Thermo Fisher	Cat# R960-25
Anti-p53	Active motif	Cat# 100,853
Mouse anti-β-actin antibody	MP Biomedicals	Cat# 691,001
Anti-human IgG (for IgG cross-linking)	MP Biomedicals	Cat# 55,087
Anti-ZTA(BZ1)	Santa Cruz	Cat# sc-53904
Rabbit IgG	Cell Signaling Tech	Cat# 2729
Halo-tag protein purification kit	VWR/Promega	Cat# PAG6790
Glutathione Sepharose 4B	GE Healthcare	Cat# 17–0756-01
Factor Xa	NEB	Cat# P8010
ChIP-Enzymatic Chromatin IP Kit	Cell Signaling Tech	Cat# 9003
Constructs		
pLX304-SAMHD1 (with PAM mutated)	Li Lab [6]	pKZ175
pLX304-SAMHD1-K469R/K595R/K622R (with PAM mutated)	This study	pSF029
pLX304	Addgene	Plasmid# 25,890
LentiCRISPR v2 vector	Addgene	plasmid# 52,961
pMD2.G	Addgene	plasmid# 12,259
psPAX2	Addgene	plasmid#12,260
pSG5-ZTA	Hayward Lab Collection	NA
Halo-HA-BGLF4	Hayward Lab Collection	pGL772
Halo-HA-UL97	Hayward Lab Collection	pGL798
Halo-V5-PIAS1	Li Lab [31]	pKZ28
pCMV-XL4-SAMHD1	Origene	Cat# SC114650
pCMV-XL4-SAMHD1-K469R	This study	pSF004
pCMV-XL4-SAMHD1-K595R	This study	pSF005
pCMV-XL4-SAMHD1-K622R	This study	pSF006
pGEX-5x-2-SAMHD1	Li lab [6]	pKZ49
pGEX-5x-2-SAMHD1-K469R	This study	pSF017
pGEX-5x-2-SAMHD1-K595R	This study	pSF018
pGEX-5x-2-SAMHD1-K622R	This study	pSF019
pGEX-5x-2-SAMHD1-K469R/K595R/K622R	This study	pSF028
Cell lines		
Akata (EBV +)	Hayward Lab Collection	NA
Akata (EBV +)-PIAS1-KO	Li Lab [31]	NA
Akata (EBV +)-SAMHD1-sg1	Li Lab [6]	NA
Akata (EBV +)-SAMHD1-sg1-pLX-SAMHD1	This study	NA
Akata (EBV +)-SAMHD1-sg1-pLX-SAMHD1- K469R/K595R/K622R	This study	NA
Akata (EBV +)-SAMHD1-sg1-pLX-Vector	Li lab [6]	NA
293 T cells	Hayward Lab Collection	NA
HEK 293 (EBV +)	[50]	NA

### Plasmids construction

Halo-BGLF4 (WT), pGEX-5x-2-SAMHD1, and Halo-UL97 (WT) were described previously [6]. Halo-V5-PIAS1, V5-PIAS1 (full length, aa 1–205, aa 1–415, aa 1–433, aa 409–651, aa 101–433, C351S) plasmids were previously described [31]. The pCMV-XL4-SAMHD1 and pGEX-5x-2-SAMHD1 were used as template to create K469R, K595R and K622R mutants using the QuikChange II site-Directed Mutagenesis Kit (Stratagene) according to the manufacturer’s instructions. Similarly, CRISPR-resistant pLX304-SAMHD1 (nucleotide G321A, silent mutation) [6] was used as a templated to create pLX304-SAMHD1-K469R/K595R/K622R using QuikChange II site-Directed Mutagenesis Kit step by step. Primers sequences used are: pSF001 (SAMHD1 K469R-F) 5’-catagtcctcccttctaatctttatttgtcctgttggctgc-3’; pSF002 (SAMHD1 K469R-R) 5’-gcagccaacaggacaaataaagattagaagggaggactatg-3’; pSF003 (SAMHD1 K595R-F) 5’-ccccactcataacacctcaaagaaaggaatggaacga-3’; pSF004 (SAMHD1 K595R-R) 5’-tcgttccattcctttctttgaggtgttatgagtgggg-3’; pSF005 (SAMHD1 K622R-F) 5’-cattgggtcatctctaaaaagctggactctgcttttgg-3’; pSF006 (SAMHD1 K622R-R) 5’- ccaaaagcagagtccagctttttagagatgacccaatg-3’.

### Lentiviral transduction of SAMHD1

To prepare lentiviruses, 293 T cells were transfected with lentiviral vector pLX304 containing the gene of WT SAMHD1, or RRR (K469R/K595R/K622R) mutant and the help vectors (pMD2.G and psPAX2; gifts from Didier Trono; Addgene plasmid #s 12,259 and 12,260) using Lipofectamine 2000 reagent. The supernatants were collected 48 h after transfection and used for infection of SAMHD1-depleted (sg1) Akata (EBV +) cells. Infected cells were selected in medium containing 2 μg/mL puromycin and 10 µg/mL blasticidin. Expression of SAMHD1 was confirmed by WB analysis.

### Cell Lysis and immunoblotting

Cells were harvested and lysed in 2 × SDS-PAGE sample buffer and boiled for 5 min. The samples were separated on 4–20% TGX gels (Cat# 4,561,096, Biorad), transferred onto PVDF membranes, and probed with primary and horseradish peroxidase-conjugated secondary antibodies.

### Protein expression and purification

Halo-tagged PIAS1, BGLF4 and UL97 proteins were expressed and purified as previously described [6, 31, 40]. Briefly, Halo-tagged plasmids were transfected into 293 T cells. Three T75 flasks of transfected cells were harvested 48 h post-transfection at 100% confluence and lysed with 2 ml HaloTag Protein Purification Buffer (50 mM HEPES pH7.5, 150 mM NaCl, 1 mM DTT, 1 mM EDTA and 0.005% NP40/IGEPAL CA-630) with Protease Inhibitor Cocktail. Halo-proteins were enriched using the Halo-tag resin and purified proteins were eluted from the resin by washing 3 times with 0.5 ml HaloTag Protein Purification Buffer containing 3µl Halo-TEV protease. The pGEX-5x-2-SAMHD1 and pGEX-5x-2-SAMHD1 mutants (K469R, K595R, K622R) proteins were purified from *E. coli*, and as described previously [6].

### Immunoprecipitation assay

293 T cells (50–60% confluence) were transfected with indicated plasmids using Lipofectamine 2000. The cells were harvested at 48 h post-transfection and lysed in RIPA lysis buffer (50 mM Tris–HCl, 150 mM NaCl, 1% NP40, 1% deoxycholate, 0.1% SDS and 1 mM EDTA) containing protease inhibitors and phosphatase cocktail I and II. The immunoprecipitation was carried out as previously described [31].

### In Vitro SUMOylation Assay

SUMO2 Conjugation Kit and SUMOlink SUMO2/3 kit were used to perform in vitro SUMOylation assay. The assay was carried out in a buffer containing 40 mM Tris pH 7.1, 40 mM NaCl, 1 mM β-ME, 5 mM MgCl2. Each protein SUMOylation reaction contained 100 nM SAE1/SAE2 (E1), 2 µM 6xHis-Ube2I/UBC9 (E2), 50 µM SUMO2, 4 mM ATP. PIAS1 was added as E3 ligase. The reaction mixtures were incubated at 37 °C for 3 h and SAMHD1 SUMOylation was analyzed by WB.

### In vitro kinase assay

In vitro kinase assay was performed as described previously [6]. Briefly, 20 ng non-SUMOylated or pre-SUMOylated SAMHD1 was incubated in 40µl Kinase Buffer containing 0.75 (v/v) magnesium-ATP cocktail buffer (Cat# 20–113; Upstate) and 6 μl of EBV BGLF4 and HCMV UL97 for 30 min at 30 °C. Finally, reaction mixtures were separated by gel electrophoresis and SAMHD1 and phospho-SAMHD1 proteins were detected by WB.

To generate phospho-SAMHD1 for in vitro SUMOylation assay, SAMHD1 was phosphorylated by EBV BGLF4 in vitro for 6 h at 4 °C as described previously [6, 17]. The buffer was included as for non-phospho-SAMHD1. The phosphorylation of SAMHD1 on T592 was confirmed by WB [6, 17].

### Lytic induction and cell treatment

To induce the EBV lytic cycle, the Akata (EBV +) cells were treated with IgG (1:200, Cat# 55,087, MP Biomedicals) for 0 and 48 h. For lytic induction of EBV in HEK293 (EBV +) cells, the cells were transfected with EBV ZTA plus other plasmids as appropriate using Lipofectamine 2000 reagent for 48 h.

### EBV DNA detection

To measure cell associated viral DNA, total genomic DNA was extracted using the Genomic DNA Purification Kit (Cat# A1120, Promega). The relative viral genome copy numbers were determined by quantitative polymerase chain reaction (qPCR) using primers specific to *BALF5* gene normalized by β*-actin* as we described previously [6].

### Structure analysis for SAMHD1

Full length SAMHD1 sequence was uploaded to the I-TASSER Protein Structure and Function Prediction tool (https://zhanglab.ccmb.med.umich.edu/I-TASSER/) [53, 54] (Roy et al. 2010; Yang et al. 2015; Zhang 2008) using default settings. Molecular graphics and analyses of tetrameric and monomeric SAMHD1 were performed with the UCSF Chimera package [55] (http://www.rbvi.ucsf.edu/chimera). Chimera is developed by the Resource for Biocomputing, Visualization, and Informatics at the University of California, San Francisco (supported by NIGMS P41-GM103311).

### Chromatin-immunoprecipitation (ChIP) assay

2 × 10^7^ WT PIAS1-KO Akata (EBV +) or cells were cross-linked with 1% formaldehyde and digested with micrococcal nuclease to achieve DNA fragments of 150–900 bp. 10% of the chromatin was reserved as input sample. DNA–protein complexes were immunoprecipitated with Anti-SAMHD1 antibody (Bethyl, Cat# A311-354) and rabbit IgG control (Cell Signaling Technology, Cat# 2729). ChIP was performed using an Enzymatic Chromatin IP kit (Cell Signaling Technology, SimpleChIP Enzymatic Chromatin IP kit) as described previously [31]. The input and ChIP samples were then reverse crosslinked and DNA was extracted and quantified by quantitative PCR (qPCR) with the specific primers for *ZTA* and *RTA* promoters (ZTAp and RTAp), and OriLyt (BHLF1p) as described previously [31].

### Quantification and statistical analysis

Statistical analyses employed a two-tailed Student’s t test. A p value of  ≤ 0.05 was considered statistically significant. Values are given as the mean of three biological replicate experiments. Error bars represent the standard deviation from three biological replicates.

## Data Availability

All data generated or analyzed during this study are included in this published article. All of the data and material in this study are available when requested.

## Abbreviations

EBV: Epstein-Barr virus
HCMV: Human cytomegalovirus
HSV-1: Human simplex virus 1
SAMHD1: Sterile alpha motif and HD domain 1
dNTPs: Deoxyribonucleoside triphosphates
dNTPase: Deoxynucleotide triphosphohydrolase
PIAS1: Protein inhibitor of activated STAT1
SIM: SUMO interacting motif
WT: Wild-type
WB: Western Blot
Co-IP: Co-immunoprecipitation
qPCR: Quantitative polymerase chain reaction
HIV-1: Human immunodeficiency virus-1
